# Supplementary material for: TCGEx: a powerful visual interface for exploring and analyzing cancer gene expression data
Source: EMBO Rep. 2025 Mar 3;26(7):1863–90. doi: 10.1038/s44319-025-00407-7 (PMC11976970; doi:10.1038/s44319-025-00407-7)
Supplement: Supplementary file 4 — Appendix [file 44319_2025_407_MOESM4_ESM.pdf]

Table of Contents

Appendix Figures.. 2

Appendix Figure-S1.. 2

Appendix Figure-S2.. 3

Appendix Figure-S3.. 4

Appendix Figure-S4.. 5

Appendix Figure-S5.. 6

Appendix Methods.. 7

Instructions for reproducing Figures 4 and 5.. 7

Figure 4A.. 7

Figure 4B.. 7

Figure 4C-D.. 7

Figure 4E.. 8

Figure 4F.. 8

Figure 4G.. 8

Figure 5A-C.. 8

Figure 5D.. 9

Figure 5E.. 9

Figure 5F-H.. 9

Figure 5I.. 9

Figure 5J.. 9

Broad Single Cell Portal Queries.. 10

SCP11:.. 10

SCP109:.. 10

SCP1493:.. 11

Original sources of the TCGEx data.. 12

Appendix Figures

Appendix Figure-S1

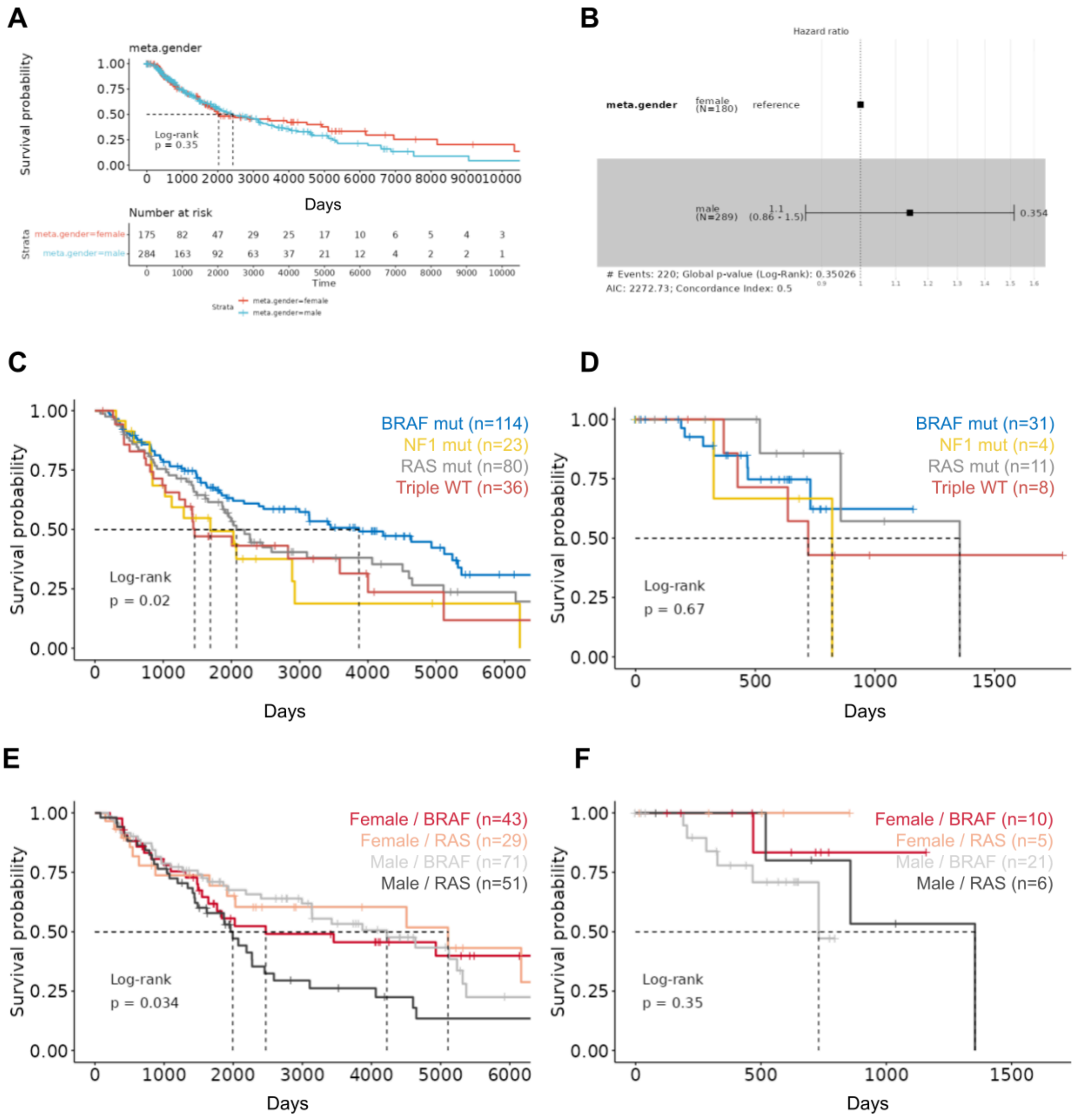

Appendix Figure S1. Modeling the effects of patient sex on melanoma survival (related to Fig.2). A. Kaplan-Meier survival analysis of sex groups in SKCM shows non-significant differences, although male patients tended to have decreased survival in longer follow-up. **B.** Cox proportional hazards survival analysis of sex effects in SKCM reveals no significant differences between the two groups. However, the male group has a slightly higher risk of death as seen in the forest plot. **C-D.** Survival curves of metastatic (C) and primary (D) melanoma are compared in SKCM. **E-F.** The relationship between survival and tumor mutational subtypes were examined for female and male patients in metastatic (E) and primary (F) SKCM samples.

Appendix Figure-S2

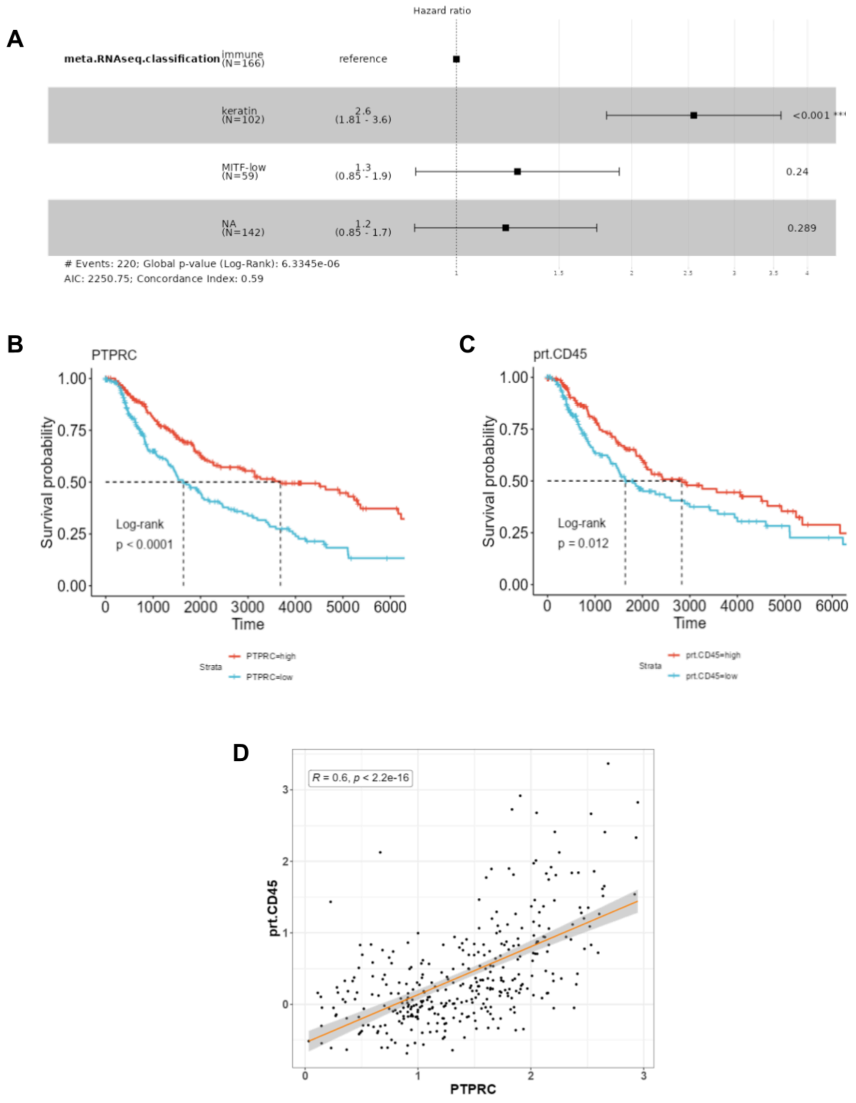

Appendix Figure S2. Survival modeling of transcriptomic subsets of melanoma (related to Fig.3). A. The forest plot shows the hazard ratios of keratin, MITF-low, and unannotated groups in comparison to the immune subset appointed as the reference group. The keratin subset is associated with a statistically significant poor outcome in melanoma compared to the immune subset. MITF-low group tended to have poorer outcomes compared to the immune subset, although this difference was not statistically significant. **B-C.** The improved survival outcomes in immune-enriched melanoma is also supported by the Kaplan-Meier analysis where the mRNA (B) and protein (C) expression of a pan-leukocyte marker CD45 (PTPRC) are categorized at the median. **D.** Protein and mRNA levels of CD45 are positively correlated in SKCM, as expected.

Appendix Figure-S3

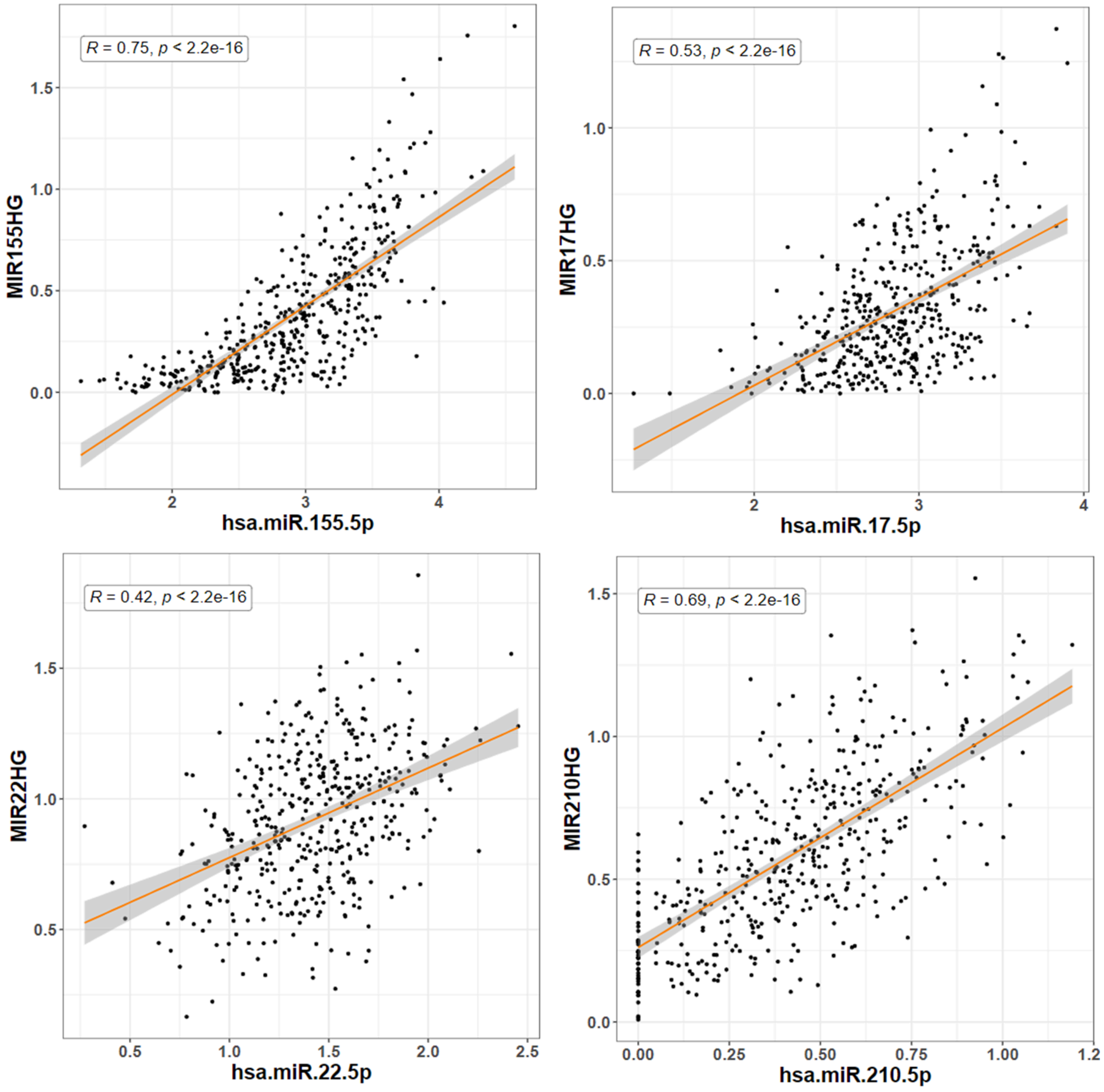

Appendix Figure S3. Comparison of the expression levels of mature miRNAs and their host genes (related to Fig.5). Scatter plots show the strong positive correlation between the host gene transcripts (RNAseq data) and the mature miRNA levels (miRNAseq data) in tumor biopsies.

Appendix Figure-S4

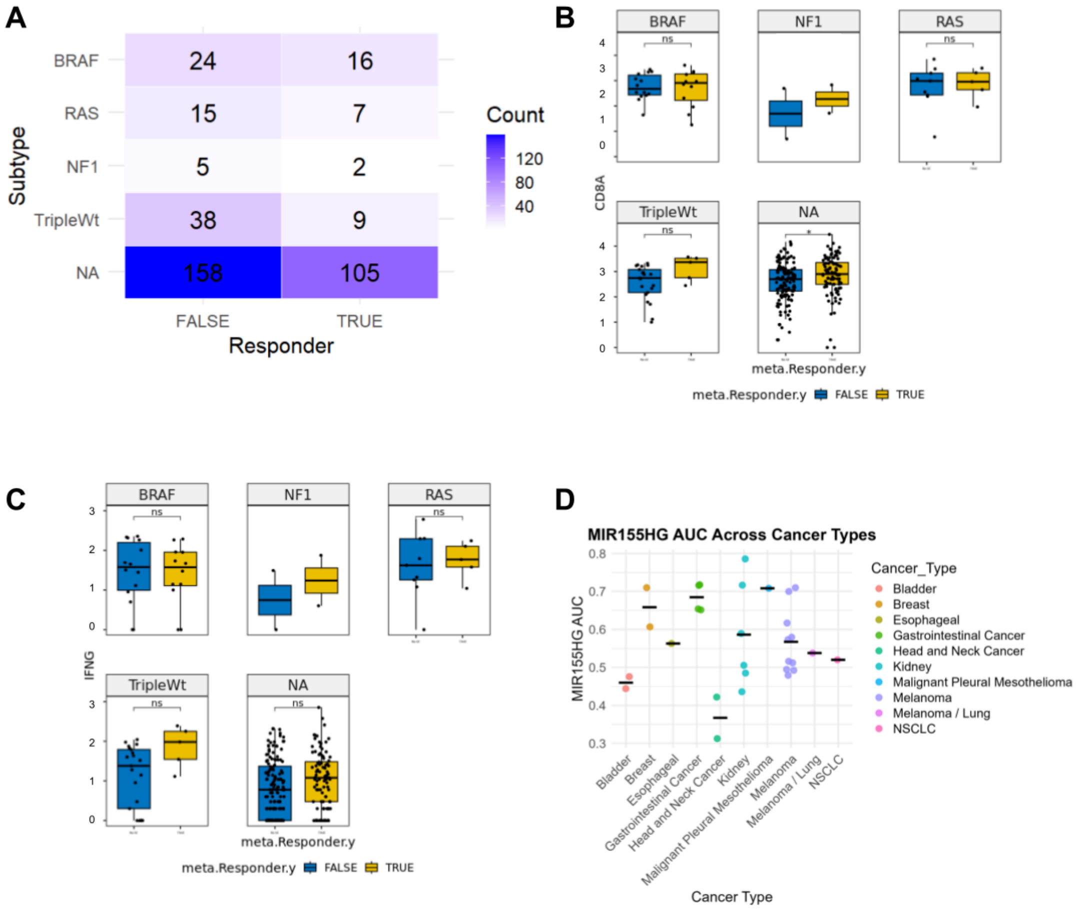

Appendix Figure S4. A. Contingency table shows the number of responding/nonresponding tumors across different mutational subsets of CRI-iAtlas melanoma data sets prior to treatment. This graph was generated using the TCGEx data object in R environment and it complements the Chi-square analyses showing no significant association between the mutational subtype and immunotherapy responsiveness in melanoma studies. However, the ratio of nonresponders to responders is relatively higher in the TripleWT subset. NA: not available. **B-C.** Immune cell-specific transcripts, CD8A (B) and IFNG (C) are plotted against immunotherapy response in different mutational subtypes of the CRI-iAtlas melanoma data sets. While small sample sizes preclude strong comparisons, immune-associated transcripts are variably expressed in melanoma mutational subtypes and between response classes. T-test p-values are shown ($p<0.0001$, "****"; $p<0.001$, "***"; $p<0.01$, "**"; $p<0.05$, "*"; $p>0.05$, "ns") **D.** AUC values of MIR155HG are plotted for different immunotherapy studies, the black lines indicate the mean AUC in different tumor types. Graph was generated in the R environment using the CRI-iAtlas pan-cancer data featured on TCGEx.

Appendix Figure-S5

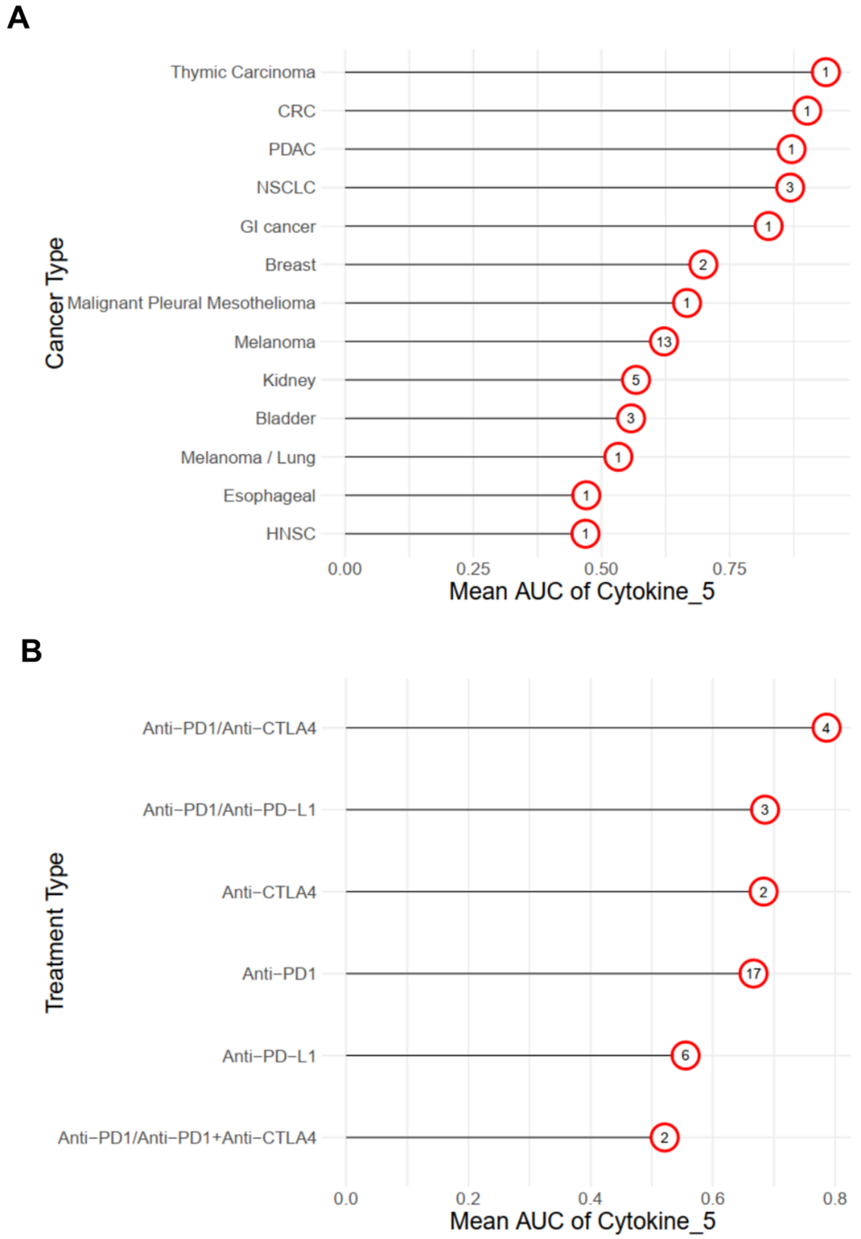

Appendix Figure S5. Examination of the Cytokine-5 signature across different cancers and immunotherapy modalities (related to Fig.7). A. The mean AUC values of Cytokine-5 signature were summarized for different cancer types in IOSig data sets. The number of individual data sets for each cancer type is shown in circles. **B.** The mean AUC values of Cytokine-5 signature were summarized across different treatment modalities. The numbers indicate the available data sets on the IOSig platform as in (A). Graphs were generated in the R environment using the exported IOSig data.

Appendix Methods

Instructions for reproducing Figures 4 and 5

Figure 4A

1. Navigate to the Data Selection module on the TCGEx interface and select the TCGA-SKCM dataset. Click Load Data to proceed.
2. Go to the Machine Learning tab and choose Metastatic and Primary Solid Tumor under the Sample Type section.
3. Under Response Variable, select the option Create a response variable from MSigDB gene sets. From the dropdown menu, navigate to MSigDB Human Collections, then Hallmark Gene Sets, and choose the MSigDB Hallmark IFNγ Response Gene Set.
4. Set the Select how to specify genes option to Use all miRNAs available on the selected data. This will populate the Response Variables and Predictor Variables in the text boxes on the right.
5. Next, move to the Ridge/Elastic Net/Lasso Regression tab. Enable Split data into train and test sets and evaluate model accuracy, and set the training set percentage to 70%.
6. Set the alpha value to 1 and click Train and Test the Model. The results will appear on the right panel.

Figure 4B

1. Access the Correlated Genes module and select the Correlation Plot tab.
2. Choose Metastatic and Primary Solid Tumor under the Sample Type section.
3. Enter IFNγ, PRF1, and GZMB, along with the five miRNAs identified in the Machine Learning analysis, in the Select Genes field.
4. Keep the default settings and select Brown-Green as the palette in the Please select a palette dropdown menu.
5. Click Analyze to generate the correlation plot.

Figure 4C-D

1. Navigate to the ROC Analysis module. Select Metastatic and Primary Solid Tumor under the Sample Type section.
2. For Figure 4C, perform the following steps:
    - In the Choose Response Variable section, select Categorical Values.
    - Choose meta.RNAseq.classification as the Categorical Data.
    - Binarize the groups by setting the Immune group as 1, and Keratin and MITF-low subtypes as 0.
    - Under Manually Enter Gene Names, select miR-155-5p.
    - Add the IFNγ Response Gene Set as a second predictor by clicking Add MSigDB Sets as a Second Predictor.

    - Click Analyze to generate the ROC curve.
3. For Figure 4D, follow the same steps as above, but in the Select Variables to Create a Custom Predictor section, add the additional four miRNAs identified alongside miR-155-5p.

Figure 4E

1. Open the GSEA module and select Metastatic and Primary Solid Tumor under the Sample Type section.
2. In the Select Feature field, enter miR-155-5p.
3. Under Please Select an MSigDB Collection, choose Curated Gene Sets (C2) and then select CP from the dropdown menu.
4. Enable the Show Top Pathway option and click Analyze to generate the results.

Figure 4F

1. Use the same settings as in Figure 4E, but instead of enabling Show Top Pathway, select Show Specific Pathway.
2. In the dropdown, choose KEGG_CYTOKINE_CYTOKINE_RECEPTOR_INTERACTION.
3. Click Analyze to display the pathway-specific results.

Figure 4G

1. Go to the PCA module and select Metastatic and Primary Solid Tumor under the Sample Type section.
2. In the Please Select Input Genes field, choose MSigDB Gene Sets.
3. Keep the default settings and select the following in sequence: Ontology Gene Sets (C5), GO, and GOBP_ADAPTIVE_IMMUNE_RESPONSE.
4. Finally, in the Please Select Feature to Annotate field, select miR-155-5p.
5. Click Analyze to generate the PCA visualization.

Figure 5A-C

1. Open the Data Selection module and find the CRI-iAtlas part from the project list.
2. Select all SKCM ICB studies and activate the Filter Data option. Set the filtering percentage to 25.
3. Navigate to the Machine Learning module.
    - Under Select Sample Type, choose all Pre-Treatment samples.
    - In the Create a Response Variable from MSigDB Gene Sets section, select Hallmark Gene Sets and then choose IFNγ Response Gene Set as the Response Variable.
    - For predictors, choose Manually Select Genes as Predictor Variables and Use All miRNAs Host Genes Available on the Selected Data.
4. Go to the Ridge/Elastic Net/Lasso Regression tab:

- Select Use 100% of the data to train a model.
- Set the alpha value to 1 and click Train the Model.
- The graphs and tables will appear on the right side.

Figure 5D

1. Open the Scatter Plot module.
2. Under Select Sample Type, choose all Pre-Treatment samples.
3. Specify the following settings:
    - X-Axis Variable: MIR155HG
    - Y-Axis Variable: CD8A
    - Change Point Size: IFNG
    - Change Point Color: PRF1
4. Check the box for Show Best Fitting Line or Curve.
5. Click Add Faceting Variable and select meta.definition column.
6. Click Analyze to generate the scatter plot.

Figure 5E

1. Open the GSEA module.
2. Under Select Sample Type, choose all Pre-Treatment samples.
3. Set the Select Feature field to MIR155HG.
4. Enable Show Specific Pathway and choose IFNγ Response Gene Set from the Hallmark Gene Sets.
5. Click Analyze to generate the plot.

Figure 5F-H

1. Repeat the steps for Figure 5A-C, but in the Data Selection module, select 10 Different ICI Studies for the analysis.
2. All subsequent steps are identical to those for Figure 5A-C.

Figure 5I

1. Repeat the steps for Figure 5E, but ensure you select PAN-ICI Pre-Treatment Data instead of SKCM-specific data.

Figure 5J

1. Repeat the steps for Figure 5E, but in the Select Feature field, select MIR155HG.
2. Under Please Select an MSigDB Collection, choose Hallmark Gene Sets.
3. Enable Show Top Pathway and click Analyze.

Broad Single Cell Portal Queries

SCP11:

- https://singlecell.broadinstitute.org/single_cell/study/SCP11/melanoma-intra-tumor-heterogeneity?genes=%2CMIR29B2CHG%2CMIR133A1HG%2CMIR155HG%2CMIR17HG%2CMIR181A1HG%2CMIR181A2HG%2CMIR1915HG%2CMIR2052HG%2CMIR205HG%2CMIR210HG%2CMIR223HG%2CMIR23AHG%2CMIR29B2CHG%2CMIR3142HG%2CMIR320B2HG%2CMIR3936HG%2CMIR4435-2HG%2CMIR4458HG%2CMIR497HG%2CMIR503HG%2CMIR9-1HG%2CMIR99AHG%2CMIRLET7BHG%2CMIR29B2C%2CMIR133A1%2CMIR155%2CMIR17%2CMIR181A1%2CMIR181A2%2CMIR1915%2CMIR2052%2CMIR205%2CMIR210%2CMIR223%2CMIR23A%2CMIR29B2C%2CMIR3142%2CMIR320B2%2CMIR3936%2CMIR4435-2%2CMIR4458%2CMIR497%2CMIR503%2CMIR9-1%2CMIR99A%2CMIRLET7B

SCP109:

- https://singlecell.broadinstitute.org/single_cell/study/SCP109/melanoma-immunotherapy-resistance?genes=%2CMIR29B2CHG%2CMIR133A1HG%2CMIR155HG%2CMIR17HG%2CMIR181A1HG%2CMIR181A2HG%2CMIR1915HG%2CMIR2052HG%2CMIR205HG%2CMIR210HG%2CMIR223HG%2CMIR23AHG%2CMIR29B2CHG%2CMIR3142HG%2CMIR320B2HG%2CMIR3936HG%2CMIR4435-2HG%2CMIR4458HG%2CMIR497HG%2CMIR503HG%2CMIR9-1HG%2CMIR99AHG%2CMIRLET7BHG%2CMIR29B2C%2CMIR133A1%2CMIR155%2CMIR17%2CMIR181A1%2CMIR181A2%2CMIR1915%2CMIR2052%2CMIR205%2CMIR210%2CMIR223%2CMIR23A%2CMIR29B2C%2CMIR3142%2CMIR320B2%2CMIR3936%2CMIR4435-2%2CMIR4458%2CMIR497%2CMIR503%2CMIR9-1%2CMIR99A%2CMIRLET7B

- https://singlecell.broadinstitute.org/single_cell/study/SCP109/melanoma-immunotherapy-resistance?genes=%2CMIR29B2CHG%2CMIR133A1HG%2CMIR155HG%2CMIR17HG%2CMIR181A1HG%2CMIR181A2HG%2CMIR1915HG%2CMIR2052HG%2CMIR205HG%2CMIR210HG%2CMIR223HG%2CMIR23AHG%2CMIR29B2CHG%2CMIR3142HG%2CMIR320B2HG%2CMIR3936HG%2CMIR4435-2HG%2CMIR4458HG%2CMIR497HG%2CMIR503HG%2CMIR9-1HG%2CMIR99AHG%2CMIRLET7BHG%2CMIR29B2C%2CMIR133A1%2CMIR155%2CMIR17%2CMIR181A1%2CMIR181A2%2CMIR1915%2CMIR2052%2CMIR205%2CMIR210%2CMIR223%2CMIR23A%2CMIR29B2C%2CMIR3142%2CMIR320B2%2CMIR3936%2CMIR4435-2%2CMIR4458%2CMIR497%2CMIR503%2CMIR9-1%2CMIR99A%2CMIRLET7B&cluster=Malignant%20cells%20from%20melanoma%20tumors&spatialGroups=--&annotation=LABELS--group--cluster&subsample=all#study-visualize

SCP1493:

- https://singlecell.broadinstitute.org/single_cell/study/SCP1493/microenvironmental-correlates-of-immune-checkpoint-inhibitor-response-in-human-melanoma-brain-metastases-revealed-by-t-cell-receptor-and-single-cell-rna-sequencing?genes=%2CMIR29B2CHG%2CMIR133A1HG%2CMIR155HG%2CMIR17HG%2CMIR181A1HG%2CMIR181A2HG%2CMIR1915HG%2CMIR2052HG%2CMIR205HG%2CMIR210HG%2CMIR223HG%2CMIR23AHG%2CMIR29B2CHG%2CMIR3142HG%2CMIR320B2HG%2CMIR3936HG%2CMIR4435-2HG%2CMIR4458HG%2CMIR497HG%2CMIR503HG%2CMIR9-1HG%2CMIR99AHG%2CMIRLET7BHG%2CMIR29B2C%2CMIR133A1%2CMIR155%2CMIR17%2CMIR181A1%2CMIR181A2%2CMIR1915%2CMIR2052%2CMIR205%2CMIR210%2CMIR223%2CMIR23A%2CMIR29B2C%2CMIR3142%2CMIR320B2%2CMIR3936%2CMIR4435-2%2CMIR4458%2CMIR497%2CMIR503%2CMIR9-1%2CMIR99A%2CMIRLET7B&cluster=UMAP%2C%20post-QC%2C%20all%20cells&spatialGroups=--&annotation=cell%20type--group--cluster&subsample=all#study-visualize

- https://singlecell.broadinstitute.org/single_cell/study/SCP1493/microenvironmental-correlates-of-immune-checkpoint-inhibitor-response-in-human-melanoma-brain-metastases-revealed-by-t-cell-receptor-and-single-cell-rna-sequencing?genes=%2CMIR29B2CHG%2CMIR133A1HG%2CMIR155HG%2CMIR17HG%2CMIR181A1HG%2CMIR181A2HG%2CMIR1915HG%2CMIR2052HG%2CMIR205HG%2CMIR210HG%2CMIR223HG%2CMIR23AHG%2CMIR29B2CHG%2CMIR3142HG%2CMIR320B2HG%2CMIR3936HG%2CMIR4435-2HG%2CMIR4458HG%2CMIR497HG%2CMIR503HG%2CMIR9-1HG%2CMIR99AHG%2CMIRLET7BHG%2CMIR29B2C%2CMIR133A1%2CMIR155%2CMIR17%2CMIR181A1%2CMIR181A2%2CMIR1915%2CMIR2052%2CMIR205%2CMIR210%2CMIR223%2CMIR23A%2CMIR29B2C%2CMIR3142%2CMIR320B2%2CMIR3936%2CMIR4435-2%2CMIR4458%2CMIR497%2CMIR503%2CMIR9-1%2CMIR99A%2CMIRLET7B&cluster=UMAP%20of%20post-QC%20CD3%2B%20T%20cells&spatialGroups=--&annotation=cluster--group--cluster&subsample=all#study-visualize

- https://singlecell.broadinstitute.org/single_cell/study/SCP1493/microenvironmental-correlates-of-immune-checkpoint-inhibitor-response-in-human-melanoma-brain-metastases-revealed-by-t-cell-receptor-and-single-cell-rna-sequencing?genes=%2CMIR29B2CHG%2CMIR133A1HG%2CMIR155HG%2CMIR17HG%2CMIR181A1HG%2CMIR181A2HG%2CMIR1915HG%2CMIR2052HG%2CMIR205HG%2CMIR210HG%2CMIR223HG%2CMIR23AHG%2CMIR29B2CHG%2CMIR3142HG%2CMIR320B2HG%2CMIR3936HG%2CMIR4435-2HG%2CMIR4458HG%2CMIR497HG%2CMIR503HG%2CMIR9-1HG%2CMIR99AHG%2CMIRLET7BHG%2CMIR29B2C%2CMIR133A1%2CMIR155%2CMIR17%2CMIR181A1%2CMIR181A2%2CMIR1915%2CMIR2052%2CMIR205%2CMIR210%2CMIR223%2CMIR23A%2CMIR29B2C%2CMIR3142%2CMIR320B2%2CMIR3936%2CMIR4435-2%2CMIR4458%2CMIR497%2CMIR503%2CMIR9-1%2CMIR99A%2CMIRLET7B&cluster=Malignant%20cell-only%20UMAP&spatialGroups=--&annotation=individual_ID--group--cluster&subsample=all#study-visualize

Original sources of the TCGEx data

- TCGA PanCancer Atlas Data:https://gdc.cancer.gov/about-data/publications/pancanatlas
- CRI ICI Data: https://www.synapse.org/Synapse:syn24200710
- CbioPortal ICI Data: https://www.cbioportal.org/
