# Supplementary material for: TCGEx: a powerful visual interface for exploring and analyzing cancer gene expression data
Source: EMBO Rep. 2025 Mar 3;26(7):1863–90. doi: 10.1038/s44319-025-00407-7 (PMC11976970; doi:10.1038/s44319-025-00407-7)
Supplement: Supplementary file 10 — Expanded View Figures [file 44319_2025_407_MOESM10_ESM.pdf]

Expanded View Figures

Figure EV1. Expression profiles of subtype-selective genes in melanoma (related to Fig. 2).

(**A**) Expression of selected genes demonstrates comparable patterns between metastatic (TM) and primary (TP) tumor samples in SKCM (TM $n = 368$, TP $n = 103$, BRAF mutants $n = 146$, NF1 mutants $n = 27$, RAS mutants $n = 91$, Triple WT $n = 44$). (**B**, **C**) Expression of previously reported genes distinguishing melanoma mutational subtypes may show sex-specific expression patterns (female $n = 180$, male $n = 291$, BRAF mutants $n = 146$, NF1 mutants $n = 27$, RAS mutants $n = 91$, Triple WT $n = 44$). (**D**) Other transcripts selectively expressed in mutational subtypes of melanoma are shown (BRAF mutants $n = 146$, NF1 mutants $n = 27$, RAS mutants $n = 91$, Triple WT $n = 44$). The box represents the interquartile range (IQR), spanning from the 25th percentile (lower bound) to the 75th percentile (upper bound). The horizontal line within the box indicates the median (50th percentile). Whiskers extend to the smallest and largest values within 1.5× IQR from the lower (Q1) and upper (Q3) quartiles, respectively. *T* test *P* values are adjusted for multiple comparisons ($P < 0.0001$, "****"; $P < 0.001$, "***"; $P < 0.01$, "**"; $P < 0.05$, "*"; $P > 0.05$, "ns").

 © The Author(s)

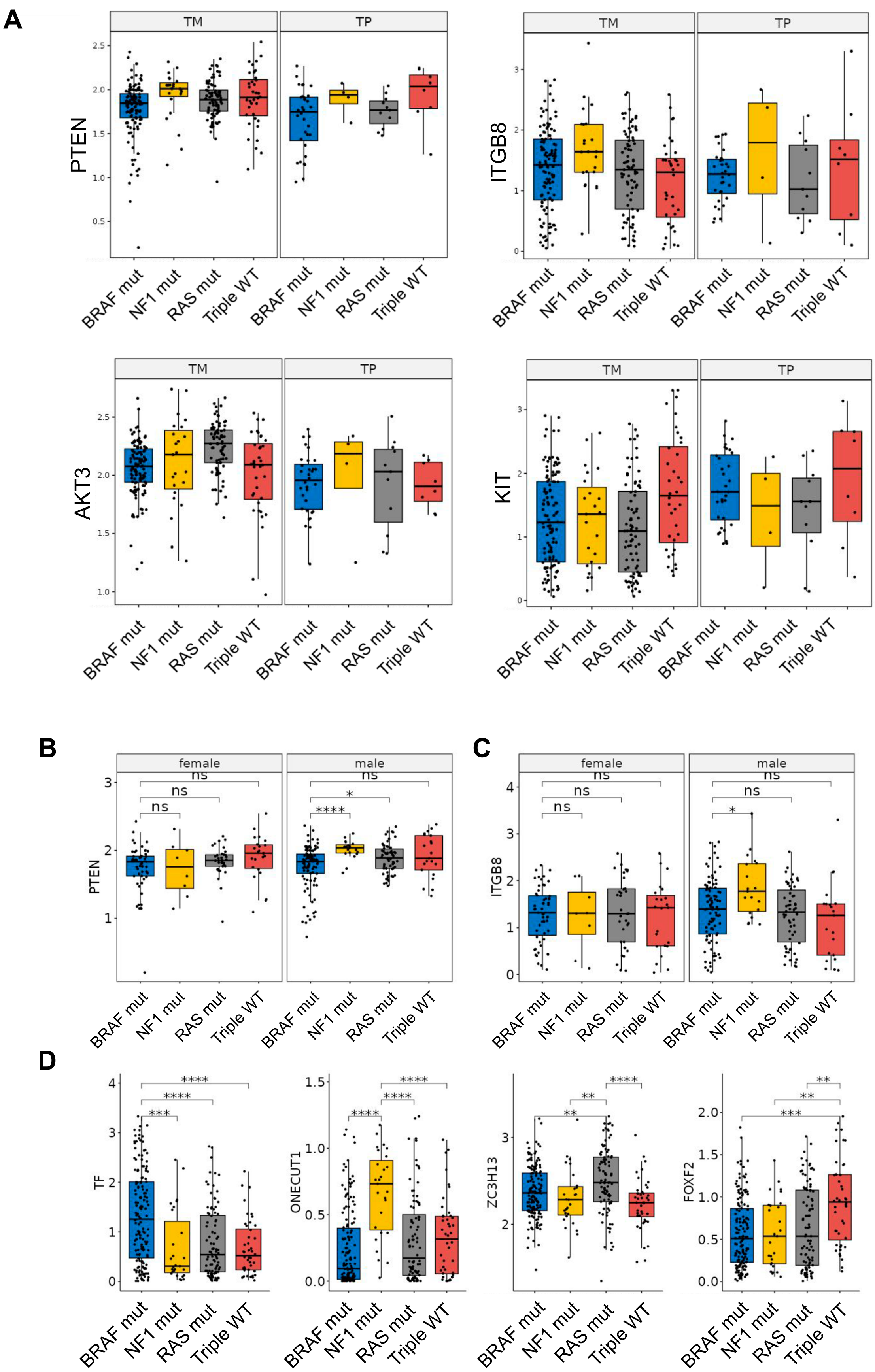
A
TM
TP
PTEN
ITGB8
AKT3
KIT
BRAF mut
NF1 mut
RAS mut
Triple WT
B
C
female
male
ns
*
****
D
TF
ONECUT1
ZC3H13
FOXF2
***
**

A

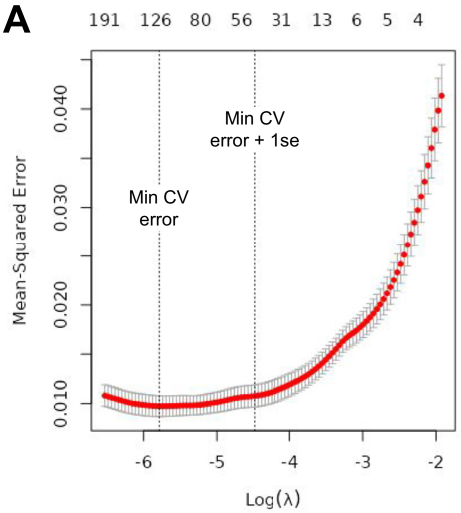

B

miRNA	Coefficient
hsa.miR.7702	0.0792
hsa.miR.3614.5p	0.0719
hsa.miR.155.5p	0.0512
hsa.miR.142.3p	0.0469
hsa.miR.5586.5p	0.0324
hsa.miR.181a.3p	0.0307
hsa.miR.100.5p	0.0266
hsa.miR.6125	0.0262
hsa.miR.503.5p	0.0260
hsa.miR.150.5p	0.0246
hsa.miR.29b.2.5p	0.0245
hsa.miR.150.3p	0.0203
hsa.miR.342.5p	0.0186
hsa.miR.4728.3p	0.0162
hsa.miR.3614.3p	0.0119
hsa.miR.181c.5p	0.0100
hsa.miR.29c.5p	0.0094
hsa.miR.342.3p	0.0080
hsa.let.7b.3p	0.0069
hsa.miR.1228.3p	0.0067
hsa.miR.542.5p	0.0057
hsa.miR.598.3p	0.0048
hsa.miR.424.3p	0.0039
hsa.miR.21.3p	0.0026
hsa.miR.30a.5p	0.0024
hsa.miR.29c.3p	0.0017

miRNA	Coefficient
hsa.miR.3170	-0.0360
hsa.miR.6509.5p	-0.0235
hsa.miR.4636	-0.0204
hsa.miR.3680.3p	-0.0191
hsa.miR.92a.3p	-0.0187
hsa.miR.340.3p	-0.0172
hsa.miR.187.3p	-0.0144
hsa.miR.19a.3p	-0.0140
hsa.miR.5699.5p	-0.0133
hsa.miR.1.3p	-0.0101
hsa.miR.6783.3p	-0.0048
hsa.miR.129.5p	-0.0047
hsa.miR.3150b.3p	-0.0045
hsa.miR.4677.3p	-0.0032
hsa.miR.219a.2.3p	-0.0029
hsa.miR.6854.5p	-0.0012
hsa.miR.144.5p	-0.0005
hsa.miR.17.3p	-0.0002

C

Positive correlation with miR-155-5p

Genes	P-value	Pearson cor. coef.
CCL5	6.59E-92	0.778
NKG7	2.24E-86	0.763
CD2	1.68E-85	0.76
TRAC	2.89E-84	0.756
TRBC2	1.87E-83	0.754
CD8A	7.18E-83	0.752
MIR155HG	9.23E-83	0.752
CD3D	1.22E-82	0.752
CD8B	1.35E-82	0.752
TIGIT	3.33E-82	0.75
UCK2	1.66E-15	-0.364
CASKIN1	1.45E-15	-0.365
SNHG29	1.02E-15	-0.367
PTK7	5.52E-16	-0.37
KCTD15	5.20E-16	-0.37
KIAA1549	3.07E-17	-0.385
GTF2IRD1	1.47E-17	-0.388
SOCS7	6.11E-18	-0.392
ADGRA3	1.88E-23	-0.448
AL031778.1	1.09E-23	-0.45

Negative correlation with miR-155-5p

D

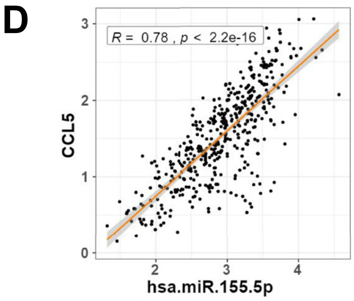

E

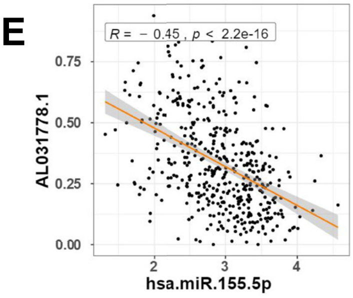

F

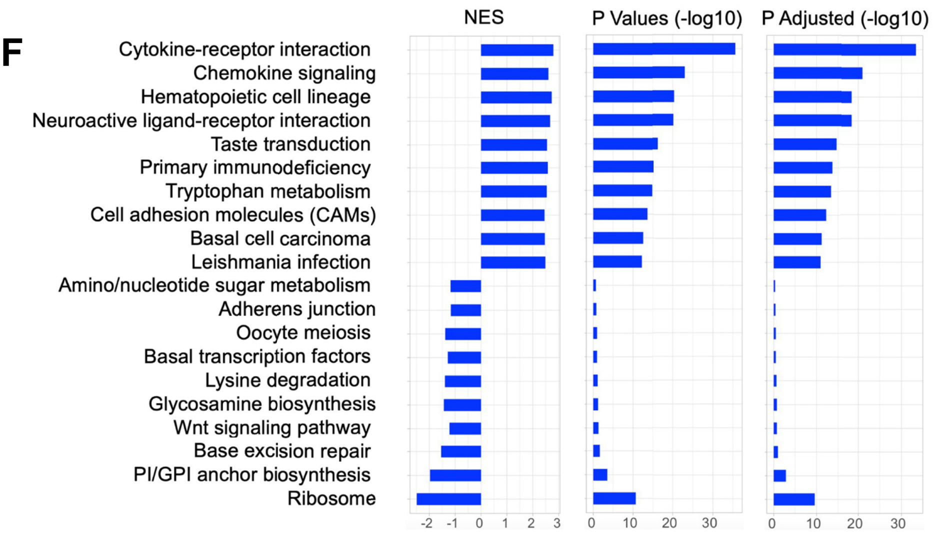

 © The Author(s)

◀ **Figure EV2. Lasso analysis of miRNAs against IFNγ signaling in melanoma (related to Fig. 4).**

(**A**) Mean-squared cross-validation (CV) error as a factor of increasing model penalty is shown ($n = 471$). (**B**) miRNA predictors with positive and negative coefficients at the lambda value corresponding to minimum CV error + 1 standard error are shown. (**C**) Transcripts with the top positive and negative correlations with miR-155-5p are shown. (**D**, **E**) TCGEx scatter plot module was used to visualize top correlators of miR-155-5p. Statistical analysis was performed using Pearson correlation test, and the corresponding *P* values are shown. (**F**) The GSEA results shown in Fig. 4E were exported from the TCGEx platform and plotted elsewhere to demonstrate that the numeric outputs can be reused by the users, if preferred.

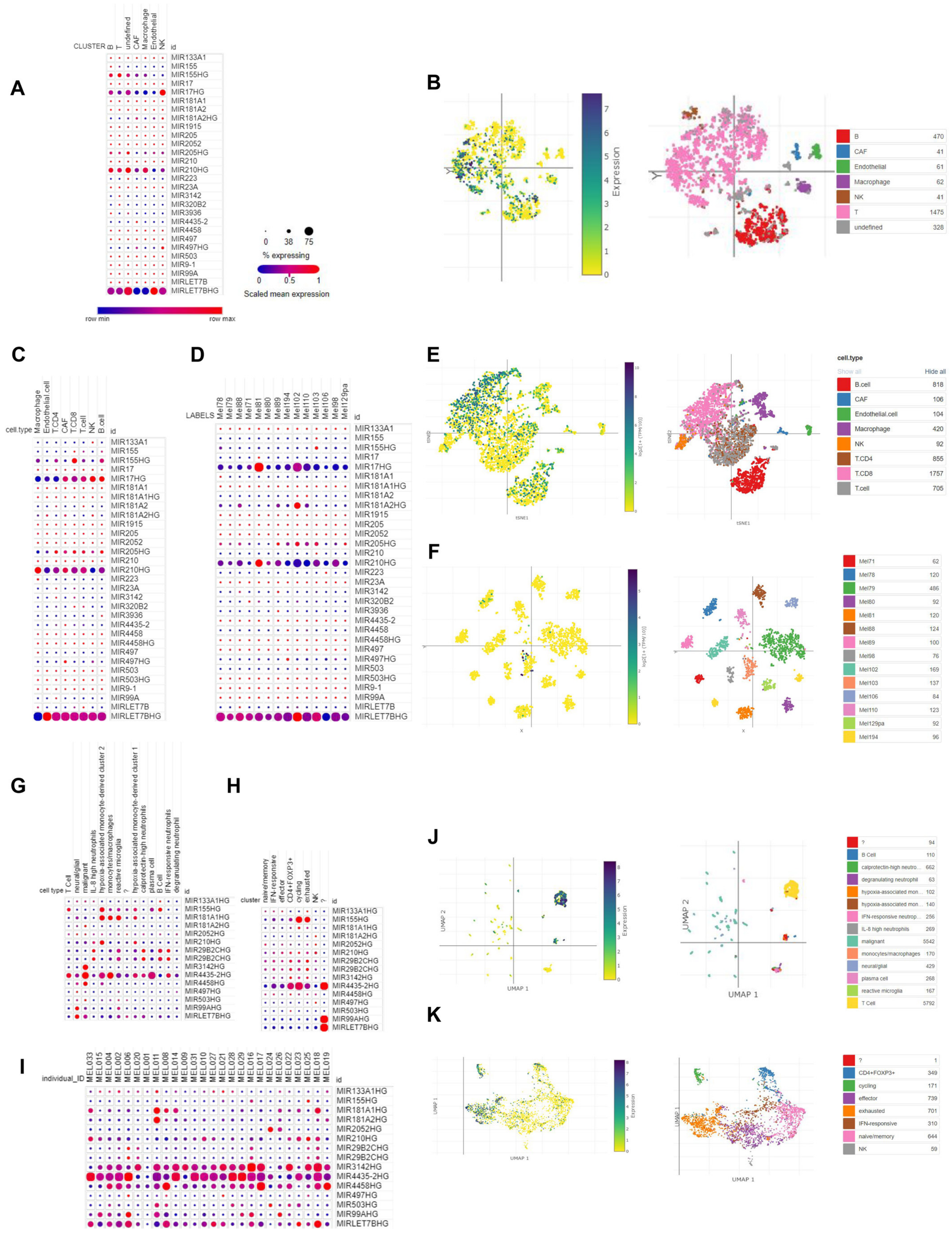
A
CLUSTER B T undefined CAF Macrophage Endothelial NK id
MIR133A1
MIR155
MIR155HG
MIR17
MIR17HG
MIR181A1
MIR181A2
MIR181A2HG
MIR1915
MIR205
MIR2052
MIR205HG
MIR210
MIR210HG
MIR223
MIR23A
MIR3142
MIR320B2
MIR3936
MIR4435-2
MIR4458
MIR497
MIR497HG
MIR503
MIR9-1
MIR99A
MIRLET7B
MIRLET7BHG
row min
row max
0 38 75
% expressing
0 0.5 1
Scaled mean expression
B
Expression
B 470
CAF 41
Endothelial 61
Macrophage 62
NK 41
T 1475
undefined 328
C
cell.type Macrophage Endothelial.cell T.CD4 CAF T.CD8 T.cell NK B.cell id
D
LABELS Mel78 Mel79 Mel88 Mel71 Mel81 Mel80 Mel89 Mel194 Mel102 Mel110 Mel103 Mel106 Mel98 Mel129pa id
MIR181A1HG
MIR4458HG
MIR503HG
E
tSNE1
tSNE2
cell.type
Show all
Hide all
B.cell 818
CAF 106
Endothelial.cell 104
Macrophage 420
NK 92
T.CD4 855
T.CD8 1757
T.cell 705
F
Mel71 62
Mel78 120
Mel79 486
Mel80 92
Mel81 120
Mel88 124
Mel89 100
Mel98 76
Mel102 169
Mel103 137
Mel106 84
Mel110 123
Mel129pa 92
Mel194 96
G
cell type T Cell neural/glial malignant IL-8 high neutrophils hypoxia-associated monocyte-derived cluster 2 monocytes/macrophages reactive microglia ? hypoxia-associated monocyte-derived cluster 1 calprotectin-high neutrophils plasma cell B Cell IFN-responsive neutrophils degranulating neutrophil id
MIR133A1HG
MIR155HG
MIR181A1HG
MIR181A2HG
MIR2052HG
MIR210HG
MIR29B2CHG
MIR3142HG
MIR4435-2HG
MIR4458HG
MIR497HG
MIR503HG
MIR99AHG
MIRLET7BHG
H
cluster naive/memory IFN-responsive effector CD4+FOXP3+ cycling exhausted NK ? id
J
UMAP 1
UMAP 2
Expression
? 94
B Cell 110
calprotectin-high neutro... 662
degranulating neutrophil 63
hypoxia-associated mon... 102
hypoxia-associated mon... 140
IFN-responsive neutrop... 256
IL-8 high neutrophils 269
malignant 5542
monocytes/macrophages 170
neural/glial 429
plasma cell 268
reactive microglia 167
T Cell 5792
K
? 1
CD4+FOXP3+ 349
cycling 171
effector 739
exhausted 701
IFN-responsive 310
naive/memory 644
NK 59
I
individual_ID MEL033 MEL015 MEL004 MEL002 MEL006 MEL020 MEL001 MEL011 MEL008 MEL014 MEL009 MEL031 MEL010 MEL027 MEL021 MEL028 MEL029 MEL016 MEL017 MEL024 MEL026 MEL022 MEL023 MEL025 MEL018 MEL019 id

Figure EV3. Publicly available scRNAseq data sets on Broad Single-Cell Portal were examined to investigate the expression patterns of ML-selected miRNAs.

Three separate data sets were analyzed: SCP11 (**A**, **B**), SCP109 (**C–F**), and SCP1493 (**G–K**); and the Single-Cell Portal interface was used to generate the plots (query URLs can be found in the Appendix). (**A**, **C**, **D**, **G–I**). Dot plots indicate the expression of miRNAs in immune (**A**, **C**, **G**, **H**) and in melanoma (**D**, **G**, **I**) cell clusters. Some miRNAs show immune cell-selective expression patterns whereas others are more specific to melanoma cells within the TME. (**B**, **E**, **F**, **J**, **K**). UMAP plots showing the expression profile of MIR155HG in immune and tumor cell clusters reveals the T and B cell-selective expression of MIR155HG within the TME (**E**, **J**). MIR155HG was particularly elevated in cycling and exhausted T-cell clusters (**K**). In contrast, melanoma cells did not broadly express MIR155HG (**F**, **J**) across three studies.

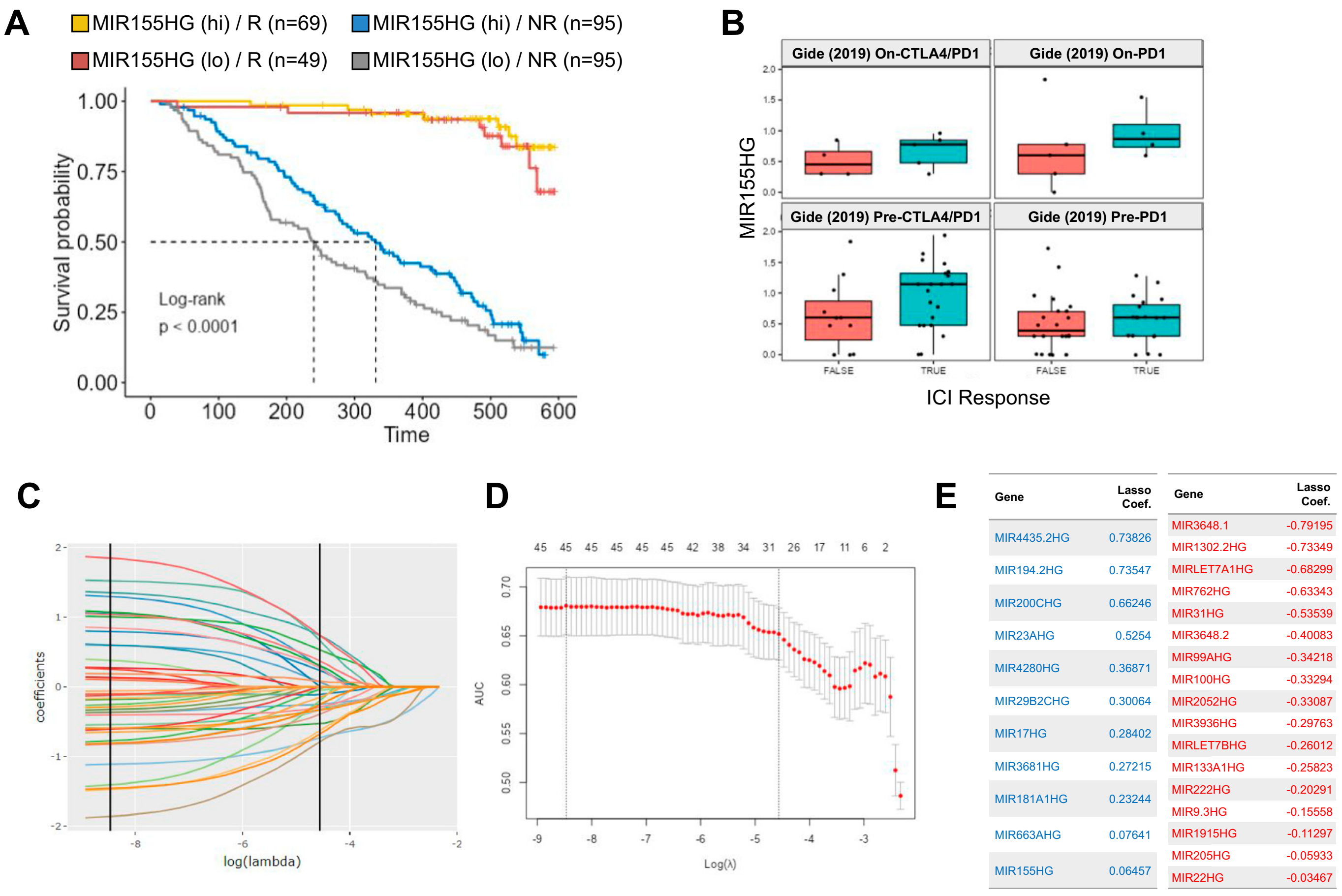

Gene	Lasso Coef.
MIR4435.2HG	0.73826
MIR194.2HG	0.73547
MIR200CHG	0.66246
MIR23AHG	0.5254
MIR4280HG	0.36871
MIR29B2CHG	0.30064
MIR17HG	0.28402
MIR3681HG	0.27215
MIR181A1HG	0.23244
MIR663AHG	0.07641
MIR155HG	0.06457

Gene	Lasso Coef.
MIR3648.1	-0.79195
MIR1302.2HG	-0.73349
MIRLET7A1HG	-0.68299
MIR762HG	-0.63343
MIR31HG	-0.53539
MIR3648.2	-0.40083
MIR99AHG	-0.34218
MIR100HG	-0.33294
MIR2052HG	-0.33087
MIR3936HG	-0.29763
MIRLET7BHG	-0.26012
MIR133A1HG	-0.25823
MIR222HG	-0.20291
MIR9.3HG	-0.15558
MIR1915HG	-0.11297
MIR205HG	-0.05933
MIR22HG	-0.03467

Figure EV4. Investigating the relationship between immunotherapy response and MIR155HG and other miRNA host genes (related to Fig. 6).

(**A**) Kaplan–Meier survival analysis demonstrates that higher MIR155HG levels (categorized at median) prior to treatment correspond to improved survival in melanoma patients ($n = 313$); however, this trend was more emphasized in non-responders. (**B**) Boxplots show the MIR155HG transcript levels in responders and non-responders during or prior to ICI treatment (On-CTLA4/PD1 $n = 9$, Pre-CTLA4/PD1 $n = 32$, On-PD1 $n = 9$, Pre-PD1 $n = 41$). The box represents the interquartile range (IQR), spanning from the 25th percentile (lower bound) to the 75th percentile (upper bound). The horizontal line within the box indicates the median (50th percentile). Whiskers extend to the smallest and largest values within 1.5× IQR from the lower (Q1) and upper (Q3) quartiles, respectively. (**C–E**) Binomial lasso was performed using binarized response (CR/PR vs PD) as response variable and 45 miRNA host genes as predictors after low-expression filtering. Regularized coefficients (**C**), the cross-validated AUC of the penalized model (**D**), and nonzero coefficients at the minimum value of lambda within 1 standard error of the highest AUC (**E**) are shown in pre-treatment melanoma patients ($n = 313$). Error bars indicate the lambda values within one standard error of the highest cross-validated AUC.

 © The Author(s)

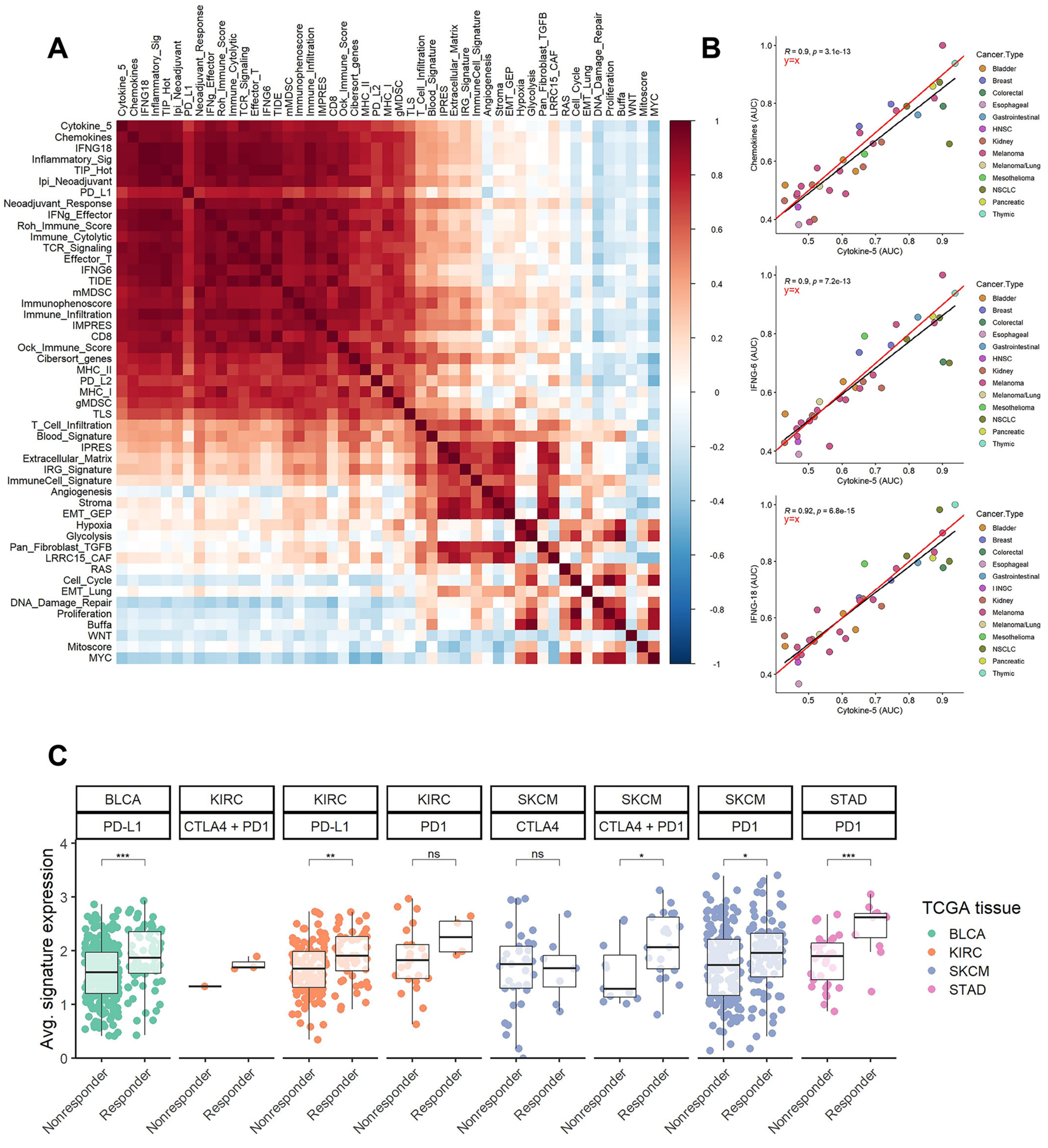
A
B
C
R = 0.9, p = 3.1e-13
y=x
Chemokines (AUC)
Cytokine-5 (AUC)
R = 0.9, p = 7.2e-13
IFNG-6 (AUC)
R = 0.92, p = 6.8e-15
IFNG-18 (AUC)
Cancer.Type
Bladder
Breast
Colorectal
Esophageal
Gastrointestinal
HNSC
Kidney
Melanoma
Melanoma/Lung
Mesothelioma
NSCLC
Pancreatic
Thymic
BLCA PD-L1
KIRC CTLA4 + PD1
KIRC PD-L1
KIRC PD1
SKCM CTLA4
SKCM CTLA4 + PD1
SKCM PD1
STAD PD1
Avg. signature expression
Nonresponder
Responder
TCGA tissue
BLCA
KIRC
SKCM
STAD

Figure EV5. Examination of the Cytokine-5 signature as a new marker for immunotherapy response (related to Fig. 7).

(**A**) The pairwise correlations between various signatures predicting immunotherapy response are depicted in a heatmap (figure was generated using the IOSig platform). The Pearson correlation coefficients were calculated by using the AUROC values across the studies in the IOSig database. (**B**) Pairwise correlations between the Cytokine-5 and IFNG6, IFN18 and Chemokine signatures are plotted in IOSig data sets. The best-fitting line (black) and its equation is shown along with the y = x diagonal line (red) indicating equality. Figure was generated in the R environment using the analysis results exported from the IOSig server. Statistical analysis was performed using Pearson correlation test, and the corresponding *P* values are shown. (**C**) The boxplots illustrate the average Cytokine-5 signature expression in immunotherapy responders and non-responders across different cancer types in TCGEx data sets (BLCA/PD-L1 $n = 348$, KIRC/CTLA4 + PD1 $n = 4$, KIRC/PD-L1 $n = 176$, KIRC/PD1 $n = 27$, SKCM/CTLA4 $n = 42$, SKCM/CTLA4 + PD1 $n = 32$, SKCM/PD1 $n = 239$, STAD $n = 45$). The box represents the interquartile range (IQR), spanning from the 25th percentile (lower bound) to the 75th percentile (upper bound). The horizontal line within the box indicates the median (50th percentile). Whiskers extend to the smallest and largest values within 1.5× IQR from the lower (Q1) and upper (Q3) quartiles, respectively. *T* test *P* values are shown ($P < 0.001$, "***"; $P < 0.01$, "**"; $P < 0.05$, "*"; $P > 0.05$, "ns")

 © The Author(s)
